# Dynamic contrast-enhanced MRI of malignant pleural mesothelioma: a comparative study of pharmacokinetic models and correlation with mRECIST criteria

**DOI:** 10.1186/s40644-019-0189-5

**Published:** 2019-02-27

**Authors:** Martina Vivoda Tomšič, Sotirios Bisdas, Viljem Kovač, Igor Serša, Katarina Šurlan Popovič

**Affiliations:** 1 Department of radiology, University Clinic of Pulmonary and Allergic Diseases Golnik, Golnik 36, Golnik, Slovenia; 2 Jozef Stefan International Postgraduate School, Jamova cesta 39, 1000 Ljubljana, Slovenia; 3 Lysholm Department of Neuroradiology, National Hospital of Neurology and Neurosurgery, London, UCLH UK; 4 Department of Radiotherapy, Institute of Oncology Ljubljana, Zaloška cesta 2, 1000 Ljubljana, Slovenia; 5 Jozef Stefan Institute, Jamova cesta 39, 1000 Ljubljana, Slovenia; 6 Institute of Radiology, University Medical Centre Ljubljana, 1000 Ljubljana, Slovenia; 7 Faculty of Medicine, University of Ljubljana, Korytkova ulica 2, 1000 Ljubljana, Slovenia

**Keywords:** Biomarker, Magnetic resonance imaging, Mesothelioma, Perfusion, Response evaluation criteria in solid tumors, Prognosis

## Abstract

**Background:**

Malignant pleural mesothelioma (MPM) is a rare and aggressive thoracic malignancy that is difficult to cure. Dynamic contrast-enhanced (DCE) MRI is a functional imaging technique used to analyze tumor microvascular properties and to monitor therapy response. Purpose of this study was to compare two tracer kinetic models, the extended Tofts (ET) and the adiabatic approximation tissue homogeneity model (AATH) for analysis of DCE-MRI and examine the value of the DCE parameters to predict response to chemotherapy in patients with MPM.

**Method:**

This prospective, longitudinal, single tertiary radiology center study was conducted between October 2013 and July 2015. Patient underwent DCE-MRI studies at three time points: prior to therapy, during and after cisplatin-based chemotherapy. The images were analyzed using ET and AATH models. In short-term follow-up, the patients were classified as having disease control or progressive disease according to modified response evaluation criteria in solid tumors (mRECIST) criteria.

Receiver operating characteristic curve analysis was used to examine specificity and sensitivity of DCE parameters for predicting response to therapy. Comparison tests were used to analyze whether derived parameters are interchangeable between the two models.

**Results:**

Nineteen patients form the study population. The results indicate that the derived parameters are not interchangeable between the models.

Significant correlation with response to therapy was found for AATH-calculated median pre-treatment efflux rate (k_ep_) showing sensitivity of 83% and specificity of 100% (AUC 0.9). ET-calculated maximal pre-treatment k_ep_ showed 100% sensitivity and specificity for predicting treatment response during the early phase of the therapy and reached a favorable trend to significant prognostic value post-therapy.

**Conclusion:**

Both models show potential in predicting response to therapy in MPM. High pre-treatment k_ep_ values suggest MPM disease control post-chemotherapy.

**Electronic supplementary material:**

The online version of this article (10.1186/s40644-019-0189-5) contains supplementary material, which is available to authorized users.

## Background

Malignant pleural mesothelioma (MPM) is a rare and aggressive thoracic malignancy that is difficult to cure. Standard treatment options include surgery, chemotherapy and radiotherapy but despite all effort, the overall survival remains poor, at 9 to 17 months [1]. There is an urgent need to improve understanding of MPM pathophysiology, and new noninvasive imaging techniques could help in tumor characterization and assessing the biophysiological effect of therapy.

The standard assessment of chemotherapy response in MPM is based on measuring tumor thickness according to modified response evaluation criteria in solid tumors (mRECIST) [2]. CT is the most widely used imaging modality for the evaluation of MPM thickness change. Despite its availability and strong clinical value, CT has limitations in differentiating subtle differences among soft tissues. MRI allows more reliable depiction of small tumor foci as well as chest wall and trans-diaphragmatic extension of MPM compared to CT. MR is also the preferred imaging method in patients allergic to iodine contrast agents [2]. Furthermore, size change due to cell death and tumor shrinkage, as a response to chemotherapy, can manifest later than changes in tumor tissue pathophysiology such as tumor vascularization and permeability. Dynamic contrast-enhanced CT (DCE-CT) and MRI (DCE-MRI) are both functional imaging techniques used to analyze tumor microvascular properties and to monitor therapy response. DCE-CT exposes patients to considerable local radiation which limits its use in research setting [3]. Therefore, DCE-MRI is the method of choice in research and in pre-clinical studies for the evaluation of early response to treatment [3]. To date, only one study has explored the potential of DCE-MRI in MPM for the assessment of therapy response with promising results [4].

In contrast to the semiquantitative methods, which allow the measurement of tracer uptake, the quantitative methods allow the calculation of perfusion parameters that are supposed to reflect the underlying physiological properties of the tissue. Transfer constant of contrast agent between the intravascular plasma compartment and extravascular interstitial compartment - *K*^trans^ is supposed to be the cardinal parameter in DCE-MRI for monitoring the therapeutic effect [5, 6]. In the present study, DCE parameters were assessed using two models: the extended Tofts (ET) model and a more complex adiabatic approximation of tissue homogeneity (AATH) model. In all models simplifying assumptions were made regarding the contrast agent kinetics and biological system [7]. Identification of the most appropriate tracer kinetic model that best describes MPM tissue pathophysiology, is of the utmost importance for the evaluation of MPM treatment response. Furthermore, the quality of the dynamic acquisitions including the temporal resolution has to be considered when selection the tracer kinetic model. To date, there is a limited number of comparative studies analyzing the effect of different tracer kinetic models for the analysis of DCE-MRI data, and none were performed in MPM [8, 9]. The ultimate goal of the investigation of this technique is the identify the pharmacodynamic endpoints that allow early assessment of MPM response to therapy.

The study had two aims. The first one was to compare two tracer kinetic models, ET and AATH for the analysis of DCE-MRI in MPM. The second aim was to examine the value of the DCE parameters to predict the response to chemotherapy in patients with MPM.

## Materials and methods

### Patients

This was a prospective study acquiring data from October 2013 until July 2015. All patients with biopsy proven malignant pleural mesothelioma eligible for chemotherapy and treated at our institution were recruited. Inclusion criteria for the study were as following: all patients have to be older than 18 years of age, have histologically proven malignant pleural mesothelioma and Karnofsky performance status ≥60% or Eastern Cooperation Oncology Group (ECOG) performance status between 0 and 2. Exclusion criteria were as follows: other malignant disease (excluding in situ cervical cancer and non-melanocytic skin cancer), acute infection, inadequate hematopoietic function (haemoglobin < 100 g/L, neutrophils < 1 × 10^9^/L, platelets < 100 × 10^9^/L), decompensated heart failure, inadequate liver function (bilirubin > 1.25 x upper normal limit, AST/ALT > 2 x upper normal limit), chronic kidney disease (glomerular filtration rate < 60 mL/min/1.73 m2), peripheral sensory neuropathy grade ≥ 2 according to Common Toxicity Criteria (CTC), vascular disorder grade ≥ 2 according to CTC, positive pregnancy test, absolute or relative contraindication to magnetic resonance imaging and gadolinium administration. The study was approved by the local ethics committee and all patients gave informed consent for participating in the study. Each patient was examined 3 times during chemotherapy with MR, including DCE-MRI: before the start of the chemotherapy, after finishing the third cycle and at the end of chemotherapy.

### Treatment schedule

The treatment schema included gemcitabine in 6-h infusion on days 1 and 8, and cisplatin at 75 mg/m2 on day 2 of 3-week cycle. After 4 cycles, patients received 2 cycles of monotherapy with gemcitabine in prolonged infusion on day 1 and 8 [10]. Patients with poor performance status received 3-weekly cycle with low-dose gemcitabine in long infusion (200 mg/m2 in 6 h on day 1) and cisplatin at 60 mg/m2 on day 2 without application on day 8 [11]. Some patient received pemetrexed and cisplatin [12].

### MR imaging protocol

The MR thoracic studies were performed by using 3 Tesla magnetic resonance system (Trio, Siemens Healthcare, Erlangen, Germany) with 6- channel body matrix coil and phase array spine matrix coil in supine position.The study started by obtaining anatomic images: T2-weighted turbo spin echo sequence with fat saturation in axial plane covering the whole thorax from clavicles to diaphragm using following parameters: repetition time [msec]/echo time [msec] 3000/99, 24 sections with an 8-mm section thickness, 1.6 mm gap, 340 × 250 mm field of view, 189 × 320 matrix, yielding a voxel resolution of 1.3 × 1.1 × 8 mm, respiratory triggered; T1-weighted tree-dimensional (3D) gradient-echo breath hold sequence (VIBE) with the following parameters: repetition time [msec]/echo time [msec], 3.18/1.15, 346 × 324 mm field of view, 1.3 × 1.1 × 1.5 mm voxel size, 246 × 320 matrix, 96 slices, 1.5 mm slice thickness, 0:39 min imaging time.DCE-MRI scans were performed over part of the thorax showing tumor burden using a T1-weighted 3D gradient echo sequence (turbo-FLASH) with following parameters: repetition time [msec]/echo time [msec], 4.5/1.16, flip angle 15°, 330 × 330 mm field of view, 192 × 192 matrix, 1.7 × 1.7 × 5 mm voxel size, 30 slices per slab with a 5 mm section thickness, with a temporal resolution of 18 s per scan. Gadolinium contrast agent (Gadovist, Gadobutrol, Berlin, Germany) was administered through the intravenous catheter after the third repetition at a dose of 0.1 mmol/kg followed by 30 ml of saline flush, both at rate of 3.5 ml/s using a power injector (Medrad, Spectris Solaris EP). Images were acquired during shallow breathing, a total of 20 sequential repetitions were acquired for 5:58 min. Prior to the dynamic image acquisition, pre-contrast T1-weighted images were acquired with 2 averages and flip angles of 2°, 10° and 15° for T1 mapping.

The study proceeded with obtaining of 3D gradient-echo breath-hold post-contrast T1-weighted images with the same parameters as the previous non-enhanced T1-weighted images.

### Imaging data analysis

All conventional and DCE-MR images were consensually analyzed by the same two radiologists with 5 and 15 years’ experience in thoracic radiology. DCE-MRI source data were post-processed on a separate workstation using commercially available software (Olea Medical 2.3, La Ciotat, France). Motion correction and signal smoothening was performed. Regions of interest (ROI) were drawn freehand around the MPM periphery in each section avoiding large vessels, readily recognizable necrotic tissue (low-attenuation nonenhancing areas within tumors), adjacent atelectasis and surrounding normal tissue.

Contrast agent concentration calculation was performed as described in reference [13].

Arterial input function (AIF) was obtained by manually selecting the aorta.

#### Tracer kinetic models

The DCE-MRI data were processed using ET and AATH models. We acquired DCE parametric maps representing transfer constant from blood plasma to extravascular, extracellular space (EES) *K*^trans^ (1/min), the volume of the blood plasma per unit volume of tissue v_p_ (ml/100 ml), the volume of EES per unit volume of tissue v_e_ (ml/100 ml), the flow rate constant between EES to plasma or efflux rate constant k_ep_ (1/min), blood flow F (ml/min/100 ml), capillary transit time TC (min) and extraction constant E (%), the area under the concentration curve AUC (mM).

#### Evaluation of the response to chemotherapy

Tumor response to therapy was assessed by same two radiologists using MR images at the first and second control study according to modified Response Evaluation Criteria in Solid Tumors (mRECIST) [14]. Patients demonstrating stable disease and partial response were classified as having disease control (DC) and patients with progressive disease as having progressive disease (PD).

### Statistical analysis

The Shapiro-Wilk test was used to test for the normal distribution of the data. If the perfusion values in the population were normally distributed parametric methods were applied. Otherwise, non-parametric paired samples test were selected for the statistical analysis.

The paired samples t test was applied to detect any statistically significant difference on the obtained DCE parameter values using both models and any difference between DCE parameters in each model.

Bland-Altman plot analysis was used to explore whether DCE parameters during the course of chemotherapy could be interchangeable between the models.

Independent samples t test was used to detect if changes in DCE parameters during the course of the therapy reflect the changes defined by the mRECIST criteria between the group of patients with DC and PD.

Receiver-operating characteristics (ROC) curve was used to examine specificity and sensibility of DCE parameters for predicting response to therapy.

The results were considered significant if *P* value < .05. *P* values were adjusted for multiple testing (Bonferroni correction).

The results were analyzed and graphs performed using MedCalc Statistical Software version 15.6.1 (MedCalc Software bvba, Ostend, Belgium; https://www.medcalc.org).

## Results

Twenty nine consecutive patients were recruited in the trial. Six patients died before the end of the chemotherapy, 2 suffered a major vascular event and discontinued the chemotherapy, 1 suffered from claustrophobia and refused further participation in the study and 1 underwent lobectomy with pleurectomy after the third cycle of chemotherapy.

Nineteen patients (16 men and 3 women, median age, 68 years, range, 46–84) formed the study population. Patient demographic and clinical data are shown in Table 1. Each patient was examined 3 times during chemotherapy with MR, including DCE-MRI: 1) at pre-treatment study (median time interval 4 days, range 7–0 days) 2) at intra-treatment study after finishing the third cycle (median time interval 8 days, range 7–14 days) and 3) at post-treatment study after finishing the sixth cycle chemotherapy (median time interval 12 days, range 7 to 14 days). Six patients were classified as having PD and 13 as DC.

**Table 1:** Demographic and clinical data

Patient	Sex/Age, years	Histology	Cycle of chemotherapy	mRECIST outcome at the end of chemotherapy	Evaluation of the response to chemotherapy
1	M/72	Epitheloid	1	PD	PD
2	F/71	Biphasic	1	PD	PD
3	M/52	Epitheloid	2	PD	PD
4	M/62	Epitheloid	1	PD	PD
5	M/73	Epitheloid	1	ST	DC
6	F/75	Epitheloid	4	PR	DC
7	M/58	Epitheloid	1	ST	DC
8	M/75	Epitheloid	1	PR	DC
9	F/46	Biphasic	1	ST	DC
10	M/61	Epitheloid	1	ST	DC
11	M/67	Sarcomatoid	2	ST	DC
12	M/60	Epitheloid	1	ST	DC
13	M/75	Epitheloid	1	ST	DC
14	M/68	Epitheloid	2	PD	PD
15	M/84	Epitheloid	1	PD	PD
16	M/60	Epitheloid	1	ST	DC
17	M/72	Epitheloid	2	ST	DC
18	M/80	Epitheloid	1	ST	DC
19	M/67	Epitheloid	4	ST	DC

*mRECIST* = modified Response Evaluation Criteria in Solid Tumors, *PD* = progressive disease, *ST* = stable disease, *PR* = partial response, *DC* = disease control

### Comparison analysis of DCE parameters between the two models

DCE parameter values differed between the two models. The v_p_ values differed in all measurements. The *K*^trans^ and v_e_ values roughly correlated. The AUC values correlated most frequently; precisely in 7 patients in pre-treatment study, 8 patients in intra-treatment study and 8 patients in post-treatment study. Detailed results are presented in Table 2.

**Table 2:** Comparison of the DCE parameters between the two models (*K*^trans^, k_ep_, AUC, v_p_, and v_e_)

	Pre-treatment study					Intra-treatment study					Post-treatment study				
Patient No.	*K* ^trans^	k_ep_	AUC	v_p_	v_e_	*K* ^trans^	k_ep_	AUC	v_p_	v_e_	*K* ^trans^	k_ep_	AUC	v_p_	v_e_
1	+	–	–	+	+	+	+	+	+	+	+	+	+	+	+
2	+	+	+	+	–	+	+	–	+	+	+	+	–	+	+
3	+	+	–	+	+	+	+	+	+	+	+	+	+	+	+
4	+	+	+	+	+	+	+	+	+	+	+	+	+	+	+
5	+	+	–	+	+	+	+	+	+	+	+	+	+	+	+
6	+	+	–	+	+	+	+	–	+	+	+	+	–	+	+
7	+	+	+	+	+	+	+	+	+	+	+	+	+	+	+
8	+	+	+	+	+	+	+	–	+	+	+	+	–	+	+
9	+	+	+	+	+	+	+	+	+	+	+	+	+	+	+
10	+	+	+	+	+	+	+	+	+	+	+	+	–	+	–
11	+	+	–	+	+	+	+	–	+	–	+	+	–	+	+
12	+	+	+	+	+	+	+	–	+	+	+	+	+	+	+
13	+	+	+	+	+	+	+	+	+	+	+	+	–	+	+
14	+	+	+	+	+	+	+	+	+	+	+	+	+	+	+
15	+	+	–	+	–	+	–	+	+	+	+	+	–	+	+
16	+	+	+	+	+	+	+	+	+	+	–	+	+	+	–
17	+	+	+	+	+	–	+	–	+	+	+	+	+	+	+
18	+	+	–	+	+	+	+	–	+	+	+	+	+	+	+
19	+	+	+	+	+	+	+	–	+	+	+	+	–	+	+

“-” indicates the *P* value > .05 and, “+” the *P* value < .05, *K*^*tran*s^ (1/min), k_ep_ (1/min), AUC (mM), v_e_ (ml/100 ml),v_p_ (ml/100 ml)

**Fig. 1:**
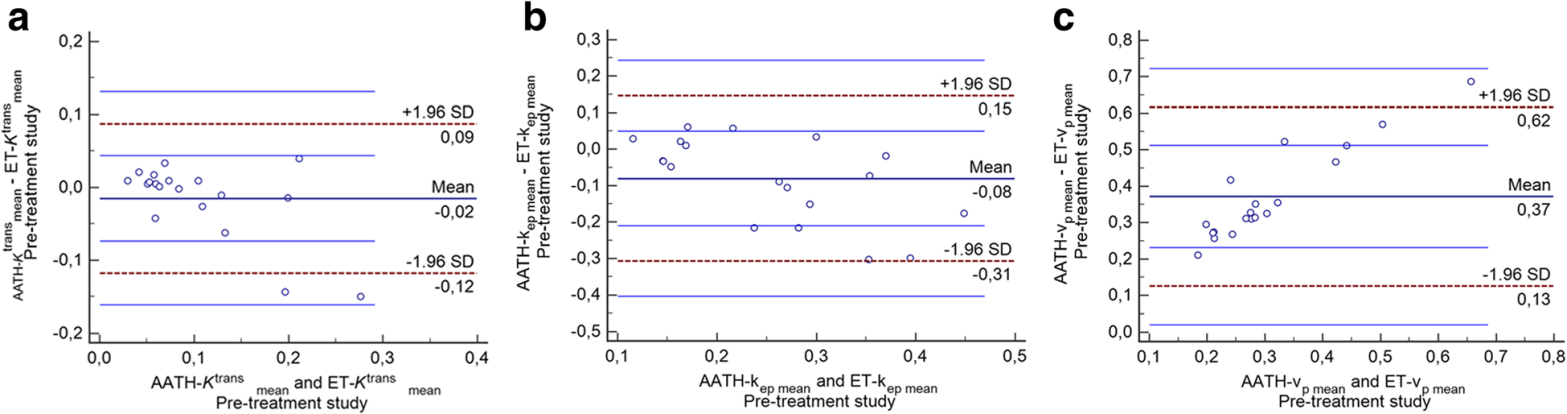
Bland-Altman agreement plot of the mean values of the DCE parameters in the pre-treatment study. Top and bottom dashes show 95% of agreement, middle line shows mean difference. **a**) transfer constant *K*^trans^ value (1/min). **b**) efflux rate constant k_ep_ values (1/min). **c**) plasma volume v_p_ values (ml/100 ml)

In Bland-Altman agreement plot, a small negative mean difference of − 0.02 (95% CI -0.04 to 0.009) was observed between the *K*^*trans*^ values with the two outliers found beyond the lower line, showing a tendency for higher bias in tumors with high permeability (Fig. 1a). A small negative difference of − 0.08 (95% CI -0.14 to − 0.02) was observed in k_ep_ values with two points found close to lower line of agreement (Fig. 1b). This shows that k_ep_ values are generally interchangeable with the caveat that in tumors with increased *k*_ep_ values, the ET model seems to overestimate the parameter relatively to the AATH.

An increasing difference between the two models was observed in v_p_ values with one outlier extending beyond the upper limit of agreement thus the v_p_ parameters are not interchangeable between the models (Fig. 1c).

### Descriptive statistics and comparison analysis of DCE parameters in both models during the chemotherapy

There was no statistically significant difference in the DCE values calculated using both models during the course of chemotherapy (see Table 3).

**Table 3:** Paired sample t test mean difference of the DCE parameters between pre- vs. intra-treatment study and between intra- vs. post-treatment study

Parameters	Intra- vs. pre-treatment study, mean +/-SD (*p*-value)	Post- vs. intra-treatment study, mean +/− SD (p-value)
ET-*K*^trans^_max_	−0.001 +/− 0.06 (>.05)	0.002 +/− 0.3 (>.05)
AATH*-K*^trans^_max_	−0.02 +/− 0.06 (>.05)	−0.06 +/− 0.2 (> 0.05)
ET*-*k_ep max_	−0.1 +/− 0.5 (>.05)	0.05 +/− 0.8 (>.05)
AATH-k_ep max_	0.1 +/− 1.6 (>.05)	- 0.2 +/− 1.7 (>.05)
ET*-*AUC _max_	− 5062 +/− 26,775 (>.05)	− 9783 +/− 29,591 (>.05)
AATH-AUC _max_	− 3310 +/− 27,804 (>.05)	− 9606 +/− 25,440 (>.05)
ET*-*v_p max_	- 0.01 +/− 0.3 (>.05)	− 0.04 +/− 0.3 (>.05)
AATH-v_p max_	0	0
ET*-*v_e med_	0.009 +/− 0.2 (>.05)	−0.05 +/− 0.3 (>.05)
AATH-v_e med_	0.07 +/− 0.2 (>.05)	− 0.09 +/− 0.4 (> 0.05)
TC-_max_	6 +/− 62. (>.05)	−4 +/− 68 (>.05)
E-_max_	0.009 +/− 0.02 (>.05)	− 0.005 +/− 0.02 (>.05)
F-_max_	−10 +/− 34 (.05)	40 +/− 168 (>.05)

Note: *K*^trans^ (1/min), k_ep_ (1/min), AUC (mM), v_e_ (ml/100 ml), v_p_ (ml/100 ml), TC (min), F (ml/min/100 ml), E (%)

The maximal ET-calculated k_ep_ values at pre-treatment study were significantly different between the group of PD and DC after the third cycle of chemotherapy (*P* = 0.01) and post-treatment (*P* = 0.04). Specifically, maximal pre-treatment ET-calculated k_ep_ values were higher by 41% in a DC group. Also, median AATH-calculated pre-treatment k_ep_ values were significantly different between the group of PD and DC post-treatment (*P* = 0.019) and were higher by 61% in DC group. Additionally, there was a similar difference when we compared mean AATH-calculated pre-treatment k_ep_ values between the group of PD and DC post-treatment (*P* = 0.015) (Additional file 1). Correlation of other values was not statistically significant.

**Fig. 2:**
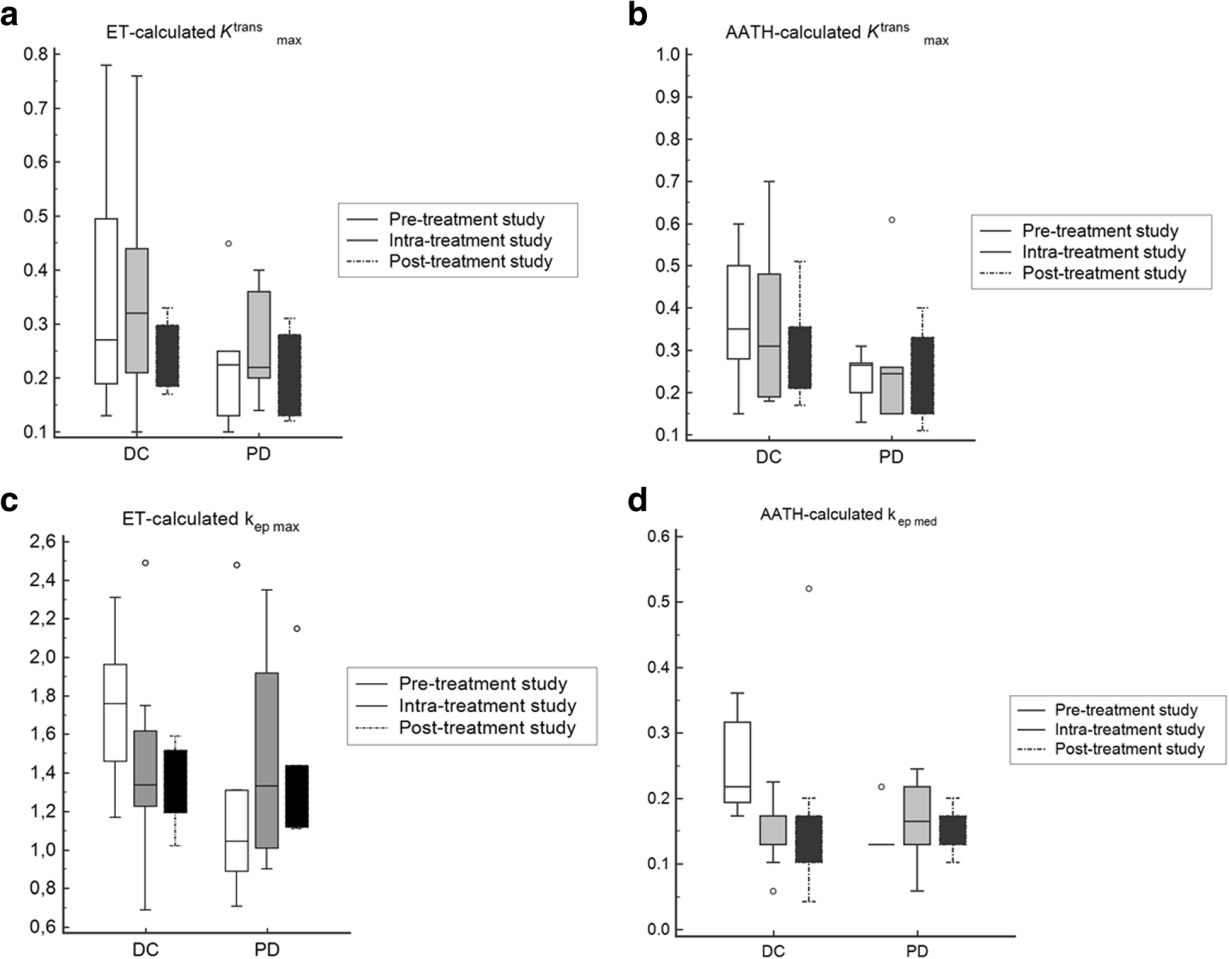
The dynamics of *K*^trans^ and k_ep_ values calculated using both models during the course of the chemotherapy. Box represents interquartile range, the line dividing the box indicates the median and circles represent the outliers. **a**) ET-calculated *K*^trans^_max_ values. **b**) AATH-calculated *K*^trans^_max_ values. **c**) ET-calculated k_ep max_ values. **d**) AATH-calculated k_ep med_ values

**Fig. 3:**
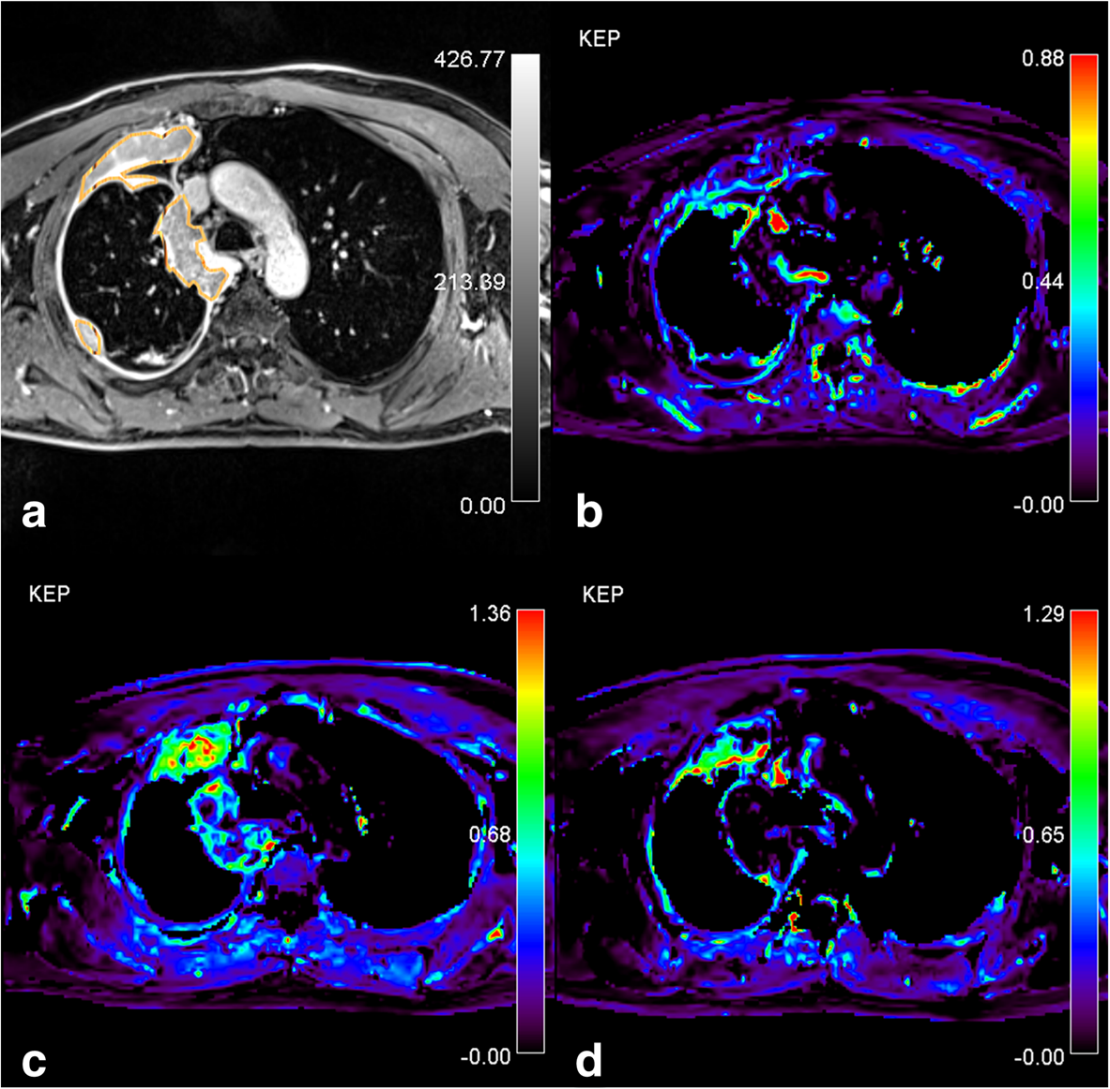
Patient with progressive disease. **a**) Post-contrast T1-weighted turbo-FLASH. Source image shows the region of interest (ROI) drawn around the tumor, AATH-calculated k_ep_ parametric map (1/min) from **b**) pre-treatment **c**) intra-treatment and **d**) post-treatment study are shown. At intra-treatment study, tumor showed an increase in k_ep_ as well as in size. At the post-treatment study, a decrease in k_ep_ and a slight increase in tumor size is seen. Significant heterogeneity of the tumor can be observed

The change in the maximal values of *K*^trans^ and k_ep_ are shown in Fig. 3. The dynamics of change in ET-calculated maximal *K*^trans^ values in DC group present a slight increase intra-treatment, followed by a pronounced decrease post-treatment. *K*^trans^ values in PD group showed a marked increase intra-treatment followed by a decrease post-treatment (Fig. 2a).

The AATH-calculated maximal *K*^trans^ values showed slight but continuous reduction during treatment in both DC and PD group (Fig. 2b).

The ET-calculated maximal k_ep_ values in DC group initially decreased, followed by a slight rebound. In PD group, the values showed increased intra-treatment, followed by a decrease post-treatment (Fig. 2c).

**Fig. 4:**
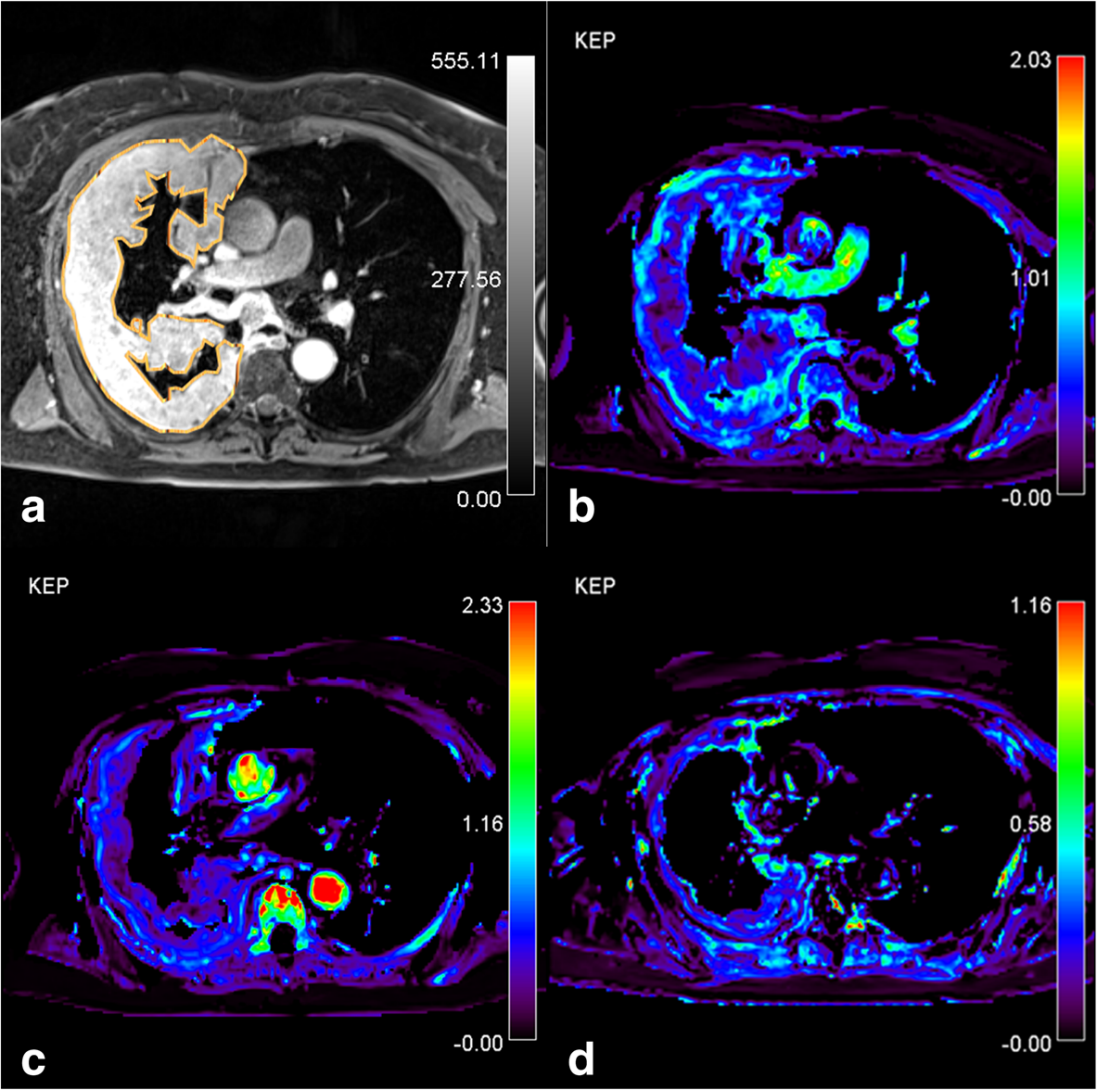
Patient with partial response. **a**) Post-contrast T1-weighted turbo-FLASH. Source image shows the region of interest (ROI) drawn around the tumor, AATH-calculated k_ep_ parametric map (1/min) from **b**) pre-treatment **c**) intra-treatment and **d**) post-treatment study are shown. Tumor reduced in size which was flowed by a reduction in the k_ep_ during the course of chemotherapy. The distribution of the k_ep_ is spatially heterogenous with high values in the periphery and low values in the core

The AATH-calculated median k_ep_ values in PD group showed an increase intra-treatment followed by a decrease post-treatment. In DC group, a persistent decrease in the values was observed (Fig. 2d). Figures 3 and 4 present k_ep_ parametric maps in two patients, one with PD and the other with DC.

AUC values using both models continuously but non-significantly decreased during the chemotherapy in DC group, whereas in PD group there was a slight increase in the first phase of the treatment, followed by a pronounced decrease in the second phase of the treatment.

### ROC curve analysis

**Fig. 5:**
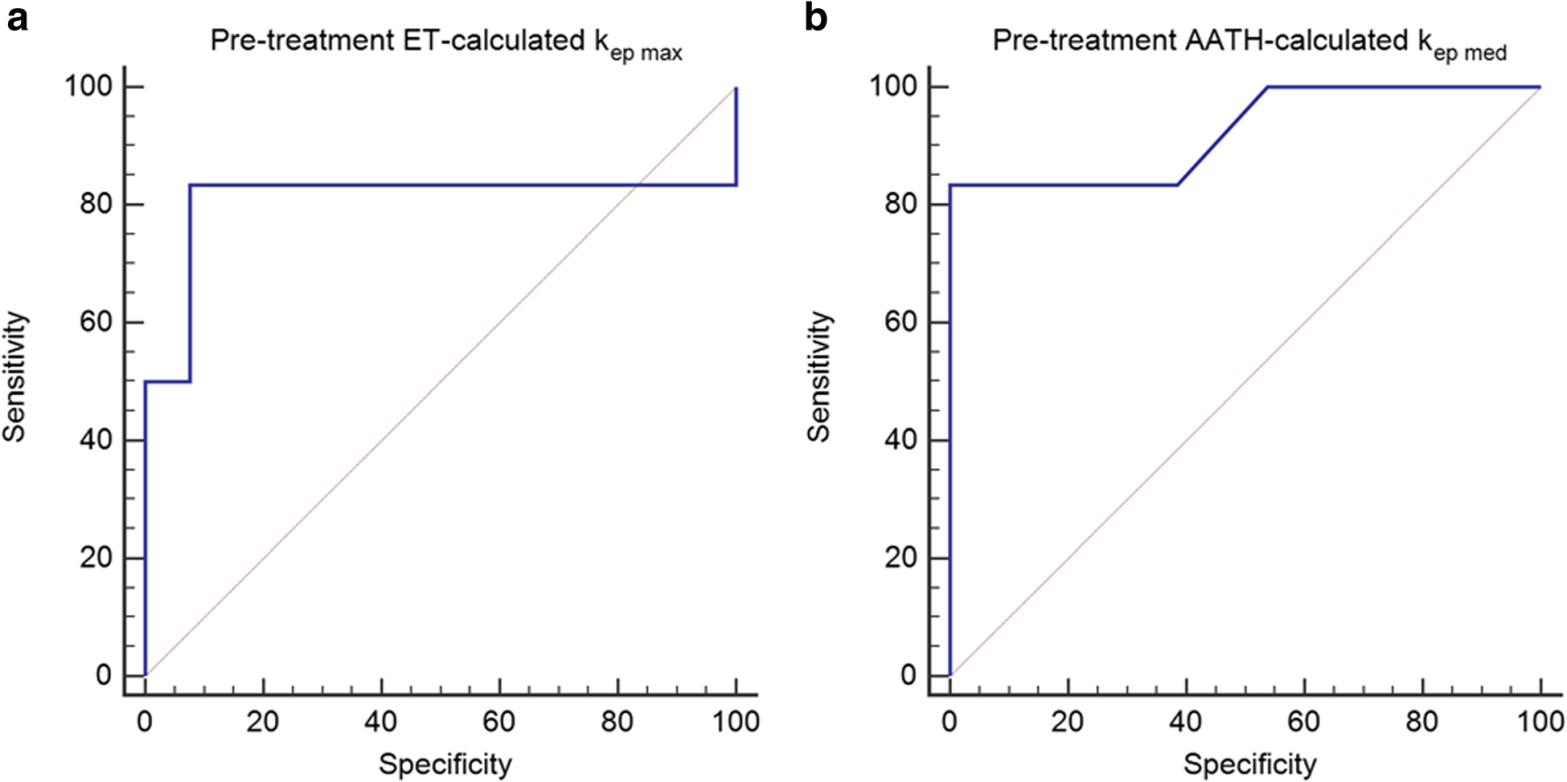
ROC of k_ep_ values for predicting tumor response to chemotherapy. **a**) ET-calculated k_ep_ values **b**) AATH-calculated k_ep_ values. Both ET- and AATH-calculated k_ep_ values show significant predictive value in predicting MPM treatment response

The ROC curve analysis showed a significant predictive value of maximal pre-treatment ET-calculated k_ep_ values for MPM response during treatment with estimated sensitivity and specificity of 100% (*P* < 0.001; cut-off of ≥0.89 /min, AUC 1). However, the maximal pre-treatment ET-calculated k_ep_ values were less predictive for the MPM response after finishing the chemotherapy with estimated sensitivity and specificity of 83.3 and 92.3% respectively (*P* = 0.06; cut-off of ≥1.31 /min, AUC 0.81) (Fig. 5a). ROC curve analysis also showed a significant predictive value of median pre-treatment AATH-calculated k_ep_ values for MPM response post-treatment with estimated sensitivity of 83,3% and specificity of 100% (*P* < 0.0001; cut-off of ≥0.13 /min, AUC 0.92) (Fig. 5b). Other DCE parameters values showed no predictive value for MPM response to therapy.

## Discussion

The crucial role of modern imaging is to map the tumor functional and imaging properties before and early during the treatment and guide the selection of the best available treatment regime and to identify early response failure prompting for second line treatment in a timely manner. Hence, the development of reliable and robust imaging biomarkers is of paramount interest in personalized tumor treatment.

Our patients were enrolled under the currently conventional treatment scheme which included cisplatin with gemicitabine or pemetrexed. Gemcitabine and pemetrexed are both cytotoxic agents, whereas cisplatin has a cytotoxic and anti-angiogenic compound [15]. It has been noted that cytostatics may exert change on tumor vasculature and interfere with angiogenic cascade without causing endothelial cell death [16, 17].

Based on this treatment effect, we were inclined to use the perfusion MRI-derived biomarkers as a surrogate for monitoring the treatment response. To start with, we attempted to address any ambiguity for the most accurate capture of the underlying pathophysiology using the current state-of-the-art DCE tracer kinetic models. ET model is broadly established because of its robustness and rather simple computational demand, but AATH presents a more elaborate though computationally demanding tracer kinetic modeling approach with the prerequisite of sufficient raw data quality. Maybe not surprisingly, quantitative DCE parameters were significantly different between the two models. Only AUC values, which is a lump quantitative parameter reflecting the complex gadolinium behavior of synchronous flow, permeability and compartmental volumes features of the tumor under investigation, correlated in around half of the patients in all studies, a finding consistent with previously published studies [7].

Nevertheless, since there is no consensus regarding the choice of the most appropriate model, a pragmatic approach to start using DCE as therapy monitoring tool is to estimate the magnitude of difference between the two broadly used models. Slight difference might be acceptable across different centers, though it is strongly recommended to use the same model within the same institution and patient surveillance, but more profound differences would flag up this caveat in future studies. Bland-Altman agreement plot analysis, an established method to detect the agreement levels between two different measurements, showed that baseline DCE parameters are satisfactory interchangeable between the two models only in tumors with fairly low perfusion and low neo-vascularity. In highly vascularized tumors, the AATH-calculated v_p_ values were notably higher compared to the ET-calculated model values and there was a tendency for increasing difference in *K*^trans^ and k_ep_ parameter values. This is consistent with the previous reported studies, where the authors suggested that ET model may underestimate v_p_ and overestimate *K*^trans^ parameter [18].

Pre-treatment ET and AATH-calculated k_ep_ values were shown to predict the treatment response during and after the chemotherapy. In this study, k_ep_ showed an interval decrease in DC group. A decrease in k_ep_ was due either to increase in v_e_ or increase in *K*^trans^ values. Both phenomena can be attributed to damages to the tumor cells as a result of cytotoxic and the concurrent anti-angiogenic action. The values of v_e_ using both models were increasing in DC group and decreasing in PD group. Cell death may reflect as an increase in v_e_ and more chaotic tissue architecture [16]. In PD group, k_ep_ values continuously increased in both models, while *K*^trans^ values were stable or slightly decreasing, reflecting persistent high permeability as a predominant angiogenic feature. In other words, though any cytostatic effect might be present, the persistent angiogenesis drives the tumor resistance to the applied therapy. This finding may suggest that adding in the therapeutic regiment an anti-angiogenic substance may reverse the unfavorable outcome. Also, the anti-angiogenic chemotherapy effect is believed to be most effective in disrupting the growth of immature new vasculature [19]. Our study population consisted of patients in all disease stages with mature vascular phenotype. Including patients only in the earlier disease stages could show the maximum therapeutic benefit of the anti-angiogenic effect.

We also evaluated the dynamics in DCE parameters during the course of chemotherapy. The results demonstrate no significant difference in DCE parameters. Opposite to the observations in other studies [19], *K*^trans^ values show a slight decline during the first phase of the treatment in both models, followed by a slight decrease in the ET model and slight increase in the AATH model in the second phase of the treatment. The physiological meaning of *K*^trans^ can be difficult to interpret in a straightforward manner. *K*^trans^ values may reflect changes in the vessel wall permeability or changes in blood flow, depending on the predominant effect (either much higher *K*^trans^ or significant high flow) with uncertainly residing in the ET model in case of similar values in these parameters [6]. During any antiangiogenic treatment, a decrease in vascular permeability is expected due to normalization of leaky neo-vessels, which can be observed as a decrease of *K*^trans^ values [19]. Our patients in DC group, ET-calculated *K*^trans^ values in the early phase of the treatment showed slight increase, followed by a decrease in the second phase of the treatment. Interval increase or stable values of K^trans^ can be found in non-responsive tumors [20, 21]. High *K*^trans^ values, in a setting where *K*^trans^ predominantly reflects blood flow, is however believed to improved drug delivery and treatment response, as observed in other tumors [22–24]. AUC values using both models continuously decreased in DC group during the chemotherapy, compared to PD group. AUC has been proposed as a primary end point alternative to K^trans^ for assessing the effect of anti-angiogenic therapy as it doesn’t require model fitting and is relatively robust [3].

A study by Giesel et al. was published on the use of DCE-MRI to assess the effect of chemotherapy in MPM where Brix model was used for analysis [4]. Similar to simplified Tofts model, plasmatic compartment in Brix model is neglected and only the v_e_ compartment is considered. Exchange rate constant between the extravascular and vascular compartment (k_ep_) in Brix model can be compared to k_ep_ parameter assessed by ET and AATH model. As in our study, responders demonstrated decrease in k_ep_ values, and non-responders demonstrated increase in k_ep_ values during treatment. Giesel et al. attributed that to the proportion of the contrast agent distribution in the tissue of interest, which is significantly higher in MPM compared to normal tissue, as in high vascularized tissue. In another study by Giesel and coworkers, immunohistochemistry was used and high microvascular density in MPM was demonstrated [25]. This further overwhelmingly justifies our choice to use the ET model, which was developed as an extension to Tofts model, as a need to account for tumor plasma volume has been recognized [26]. Third calculated parameter using Brix model (k_el_) does not allow extrapolation to any parameter assessed with either ET or AATH model.

The first and main limitation, in our opinion, is the relatively small number of the patients. This reflects the small incidence of MPM and on top of that not all patients were eligible for chemotherapy due to comorbidity or advanced disease stage. Despite this, the number of patients that have completed the chemotherapy and were scanned is almost three times higher, compared to the only previously published study on the use of DCE-MRI in MPM [4]. Secondly, because of the presence of diffuse pleural growth combined with small tumor foci dictated an extended anatomical coverage in our DCE-MRI experiments limiting the spatial and temporal resolution. Consequently, we chose the most suitable trade-off in order to acquire images with sufficient quality for the subsequent modelling. Reportedly, ET model analysis performs better in relatively low temporal resolution conditions [27]. Thirdly, despite the extended anatomical coverage, not all tumor foci were included in DCE-MRI scans, therefore, tumor heterogeneity was not fully accounted for. Finally, our endpoints did not include progression and overall survival time.

## Conclusion

Currently, performance status of the patients with MPM is the most important factor for selecting treatment options [2]. The results of our study showed that ET and AATH models for post-processing DCE-MRI datasets yield significantly different values of the DCE parameters. However, high pre-treatment k_ep_ values, calculated using both models, may serve as a prognostic marker in predicting better MPM response to chemotherapy. Also, a decrease in k_ep_ and AUC values was detected during the course of chemotherapy in patients demonstrating stable disease and partial response according to mRECIST. Therefore, the use DCE-MRI may open up the possibility for patient-tailored treatment depending on tumor biology [2, 28]. Furthermore, change in tumor permeability characteristics may provide different prospective of the treatment effect other than change in tumor thickness.

## Additional file

Additional file 1: **Table S1.** Mean and SD values of DCE parameters (*K*^trans^, k_ep_). **Table S2.** Paired sample ttest of the mean values of DCE parameters (*K*^trans^, k_ep_) between disease control and progressive disease group. Description of data: Mean and standard deviation values of *K*^trans^ and k_ep_ parameter calculated using both models for each measurement are listed in Table 1. Comparison of the mean values of *K*^trans^ and k_ep_ parameter calculated using both models between progressive disease and disease control group is shown in Table 2. (DOCX 29 kb)

## Abbreviations

3D: tree-dimensional
AATH: Adiabatic approximation tissue homogeneity
AIF: Arterial input function
Amp: Distribution volume of the contrast agent in the tissue of interest
AUC: Area under the concentration curve
CTC: Common toxicity criteria
DC: Disease control
DCE: Dynamic contrast enhanced
DCE-CT: Dynamic contrast-enhanced CT
DCE-MRI: Dynamic contrast-enhanced MRI
E: Extraction constant
EES: Extravascular, extracellular space
ET: Extended Toft
F: Blood flow
k_ep_: Flow rate constant between EES to plasma
*K*^trans^: Transfer constant from blood plasma to extravascular, extracellular space
MPM: Malignant pleural mesothelioma
mRECIST: Modified response evaluation criteria in solid tumors
PD: Progressive disease
ROC: Receiver-operating characteristics
ROI: Region of interest
TC: capillary transit time
v_e_: volume of EES per unit volume of tissue
v_p_: volume of the blood plasma per unit volume of tissue
