# Supplementary material for: Dynamic contrast-enhanced MRI of malignant pleural mesothelioma: a comparative study of pharmacokinetic models and correlation with mRECIST criteria
Source: Cancer Imaging. 2019 Feb 27;19:10. doi: 10.1186/s40644-019-0189-5 (PMC6391827; doi:10.1186/s40644-019-0189-5)
Supplement: Supplementary file 1 — Table S1. Mean and SD values of DCE parameters (Ktrans, kep). Table S2. Paired sample ttest of the mean values of DCE parameters (Ktrans, kep) between disease control and progressive disease group. Description of data: Mean and standard deviation values of Ktrans and kep parameter calculated using both models for each measurement are listed in Table 1. Comparison of the mean values of Ktrans and kep parameter calculated using both models between progressive disease and disease control group is shown in Table 2. (DOCX 29 kb) [file 40644_2019_189_MOESM1_ESM.docx]

**Additional file: Table 1: Mean and SD values of DCE parameters (*K*^trans^, k_ep_)**

|  | | **Pre-treatment study** | | | | **Intra-treatment study** | | | | **Post-treatment study** | | | |
| --- | --- | --- | --- | --- | --- | --- | --- | --- | --- | --- | --- | --- | --- |
| **Patient No.** |  | **ET- *K*^trans^** | **AATH *K*^trans^** | **ET- k_ep_** | **AATH- k_ep_** | **ET- *K*^trans^** | **AATH- *K*^trans^** | **ET- k_ep_** | **AATH- k_ep_** | **ET- *K*^trans^** | **AATH- *K*^trans^** | **ET- k_ep_** | **AATH- k_ep_** |
| 1 | Mean | 0.14 | 0.13 | 0.32 | 0.23 | 0.10 | 0.12 | 0.22 | 0.22 | 0.13 | 0.06 | 0.42 | 0.18 |
|  | Std. Deviation | 0.08 | 0.08 | 0.19 | 0.15 | 0.06 | 0.07 | 0.17 | 0.16 | 0.07 | 0.04 | 0.27 | 0.18 |
| 2 | Mean | 0.06 | 0.07 | 0.19 | 0.23 | 0.11 | 0.08 | 0.26 | 0.16 | 0.15 | 0.11 | 0.41 | 0.25 |
|  | Std. Deviation | 0.04 | 0.06 | 0.16 | 0.36 | 0.05 | 0.05 | 0.18 | 0.18 | 0.07 | 0.08 | 0.23 | 0.31 |
| 3 | Mean | 0.07 | 0.07 | 0.18 | 0.17 | 0.04 | 0.03 | 0.14 | 0.13 | 0.03 | 0.02 | 0.14 | 0.14 |
|  | Std. Deviation | 0.05 | 0.05 | 0.15 | 0.20 | 0.03 | 0.03 | 0.17 | 0.18 | 0.02 | 0.02 | 0.15 | 0.18 |
| 4 | Mean | 0.05 | 0.06 | 0.14 | 0.15 | 0.13 | 0.06 | 0.60 | 0.25 | 0.04 | 0.05 | 0.25 | 0.28 |
|  | Std. Deviation | 0.03 | 0.05 | 0.14 | 0.17 | 0.07 | 0.05 | 0.32 | 0.21 | 0.05 | 0.05 | 0.29 | 0.33 |
| 5 | Mean | 0.34 | 0.20 | 0.55 | 0.27 | 0.18 | 0.11 | 0.37 | 0.18 | 0.15 | 0.12 | 0.26 | 0.22 |
|  | Std. Deviation | 0.13 | 0.09 | 0.23 | 0.16 | 0.07 | 0.05 | 0.17 | 0.15 | 0.10 | 0.08 | 0.19 | 0.18 |
| 6 | Mean | 0.23 | 0.20 | 0.53 | 0.38 | 0.20 | 0.09 | 0.40 | 0.15 | 0.06 | 0.07 | 0.23 | 0.17 |
|  | Std. Deviation | 0.15 | 0.12 | 0.27 | 0.22 | 0.16 | 0.06 | 0.26 | 0.16 | 0.05 | 0.06 | 0.19 | 0.24 |
| 7 | Mean | 0.11 | 0.11 | 0.41 | 0.34 | 0.09 | 0.37 | 0.30 | 0.38 | 0.10 | 0.08 | 0.36 | 0.30 |
|  | Std. Deviation | 0.06 | 0.07 | 0.25 | 0.26 | 0.06 | 0.21 | 0.17 | 0.22 | 0.05 | 0.06 | 0.24 | 0.30 |
| 8 | Mean | 0.08 | 0.13 | 0.34 | 0.64 | 0.07 | 0.08 | 0.18 | 0.20 | 0.05 | 0.03 | 0.21 | 0.16 |
|  | Std. Deviation | 0.08 | 0.13 | 0.32 | 0.91 | 0.05 | 0.07 | 0.15 | 0.29 | 0.04 | 0.03 | 0.20 | 0.22 |
| 9 | Mean | 0.06 | 0.07 | 0.19 | 0.24 | 0.14 | 0.05 | 0.38 | 0.10 | 0.12 | 0.10 | 0.28 | 0.21 |
|  | Std. Deviation | 0.05 | 0.05 | 0.18 | 0.26 | 0.08 | 0.04 | 0.20 | 0.11 | 0.07 | 0.07 | 0.17 | 0.34 |
| 10 | Mean | 0.09 | 0.09 | 0.32 | 0.28 | 0.17 | 0.16 | 0.27 | 0.19 | 0.10 | 0.09 | 0.25 | 0.19 |
|  | Std. Deviation | 0.06 | 0.06 | 0.23 | 0.34 | 0.10 | 0.09 | 0.16 | 0.15 | 0.04 | 0.05 | 0.17 | 0.27 |
| 11 | Mean | 0.05 | 0.06 | 0.26 | 0.34 | 0.01 | 0.02 | 0.05 | 0.13 | 0.02 | 0.03 | 0.15 | 0.16 |
|  | Std. Deviation | 0.06 | 0.06 | 0.26 | 0.42 | 0.02 | 0.04 | 0.09 | 0.33 | 0.03 | 0.05 | 0.18 | 0.31 |
| 12 | Mean | 0.29 | 0.30 | 0.43 | 0.41 | 0.07 | 0.13 | 0.18 | 0.44 | 0.15 | 0.14 | 0.30 | 0.23 |
|  | Std. Deviation | 0.26 | 0.25 | 0.36 | 0.41 | 0.07 | 0.14 | 0.21 | 0.74 | 0.10 | 0.09 | 0.21 | 0.20 |
| 13 | Mean | 0.08 | 0.09 | 0.20 | 0.26 | 0.18 | 0.15 | 0.48 | 0.34 | 0.22 | 0.04 | 0.60 | 0.09 |
|  | Std. Deviation | 0.05 | 0.07 | 0.16 | 0.46 | 0.11 | 0.13 | 0.32 | 0.44 | 0.16 | 0.05 | 0.37 | 0.16 |
| 14 | Mean | 0.13 | 0.05 | 0.41 | 0.17 | 0.15 | 0.06 | 0.52 | 0.17 | 0.05 | 0.05 | 0.18 | 0.17 |
|  | Std. Deviation | 0.12 | 0.05 | 0.36 | 0.22 | 0.11 | 0.04 | 0.33 | 0.25 | 0.03 | 0.03 | 0.18 | 0.21 |
| 15 | Mean | 0.06 | 0.06 | 0.22 | 0.23 | 0.13 | 0.10 | 0.34 | 0.22 | 0.10 | 0.10 | 0.24 | 0.18 |
|  | Std. Deviation | 0.04 | 0.04 | 0.23 | 0.27 | 0.07 | 0.07 | 0.23 | 0.27 | 0.05 | 0.06 | 0.16 | 0.18 |
| 16 | Mean | 0.03 | 0.04 | 0.17 | 0.22 | 0.03 | 0.05 | 0.10 | 0.22 | 0.08 | 0.14 | 0.43 | 0.62 |
|  | Std. Deviation | 0.03 | 0.03 | 0.15 | 0.19 | 0.03 | 0.05 | 0.16 | 0.22 | 0.06 | 0.10 | 0.29 | 0.55 |
| 17 | Mean | 0.16 | 0.13 | 0.43 | 0.30 | 0.06 | 0.07 | 0.22 | 0.21 | 0.08 | 0.06 | 0.24 | 0.16 |
|  | Std. Deviation | 0.12 | 0.11 | 0.32 | 0.31 | 0.04 | 0.06 | 0.19 | 0.26 | 0.06 | 0.05 | 0.19 | 0.20 |
| 18 | Mean | 0.18 | 0.12 | 0.45 | 0.23 | 0.19 | 0.10 | 0.45 | 0.24 | 0.10 | 0.09 | 0.68 | 0.22 |
|  | Std. Deviation | 0.09 | 0.09 | 0.27 | 0.29 | 0.10 | 0.10 | 0.27 | 0.40 | 0.09 | 0.08 | 0.35 | 0.51 |
| 19 | Mean | 0.14 | 0.15 | 0.39 | 0.42 | 0.10 | 0.12 | 0.45 | 0.41 | 0.11 | 0.12 | 0.47 | 0.38 |
|  | Std. Deviation | 0.08 | 0.08 | 0.22 | 0.38 | 0.06 | 0.06 | 0.21 | 0.28 | 0.08 | 0.07 | 0.23 | 0.26 |

Units: *K*^trans^ (1/min), k_ep_ (1/min).

| **Table 2: Paired sample t test of the mean values of DCE parameters (*K*^trans^, k_ep_) between disease control and progressive disease group** | | | |
| --- | --- | --- | --- |
| **Perfusion parameter** | **Disease control group** | **Progressive disease group** | **p-value** |
| **Pre-treatment study** |  |  |  |
| ET-*K*^trans^ _mean_ | 0.12 | 0.08 | 0.54 |
| AATH*-K*^trans^ _mean_ | 0.12 | 0.07 | 0.23 |
| ET*-*k_ep mean_ | 0.33 | 0.24 | 0.19 |
| AATH*-*k_ep mean_ | 0.33 | 0.20 | **0.015** |
|  |  |  |  |
| **Intra-treatment study** |  |  |  |
| ET-*K*^trans^ _mean_ | 0.10 | 0.11 | 0.36 |
| AATH*-K*^trans^ _mean_ | 0.12 | 0.08 | 0.21 |
| ET*-*k_ep mean_ | 0.28 | 0.35 | 0.31 |
| AATH*-*k_ep mean_ | 0.26 | 0.19 | 0.35 |
| **Post-treatment study** |  |  |  |
| ET-*K*^trans^ _mean_ | 0.10 | 0.08 | 0.82 |
| AATH*-K*^trans^ _mean_ | 0.08 | 0.07 | 0.58 |
| ET*-*k_ep mean_ | 0.36 | 0.27 | 0.76 |
| AATH*-*k_ep mean_ | 0.25 | 0.20 | 0.82 |

Note: the statistically significant value (<.05) is set in bold. Units: *K*^trans^ (1/min), k_ep_ (1/min).
